# Identification of *UBA7* expression downregulation in myelodysplastic neoplasm with *SF3B1* mutations

**DOI:** 10.1038/s41598-025-95738-9

**Published:** 2025-03-29

**Authors:** Sael Alatawi, Othman R. Alzahrani, Fuad A. Alatawi, Ibrahim A. Almazni, Alhomidi Almotiri, Fahad M. Almsned

**Affiliations:** 1 Department of Medical Laboratory Technology, Faculty of Applied Medical Sciences, University of Tabuk, 47713 Tabuk, Saudi Arabia; 2 Institute of Cancer and Genomic Sciences, University of Birmingham, Birmingham, UK; 3 Innovation and Entrepreneurship Center, University of Tabuk, 47713 Tabuk, Saudi Arabia; 4 Department of Biology, Faculty of Sciences, University of Tabuk, 71491 Tabuk, Saudi Arabia; 5 Genome and Biotechnology Unit, Faculty of Sciences, University of Tabuk, 71491 Tabuk, Saudi Arabia; 6 Department of Clinical Laboratory Sciences, Faculty of Applied Medical Sciences, Najran University, Najran, Saudi Arabia; 7 Department of Clinical Laboratory Sciences, College of Applied Medical Sciences, Shaqra University, Shaqra, Saudi Arabia; 8 European Cancer Stem Cell Research Institute, School of Biosciences, Cardiff University, Cardiff, UK; 9 Research Program, Academic, Training, and Research Administration, Eastern Health Cluster, Dammam, Saudi Arabia; 10 Research Center, King Fahad Specialist Hospital in Dammam, Dammam, Saudi Arabia; 11 School of Systems Biology, George Mason University, Fairfax, VA USA; 12 Department of Research and Development, Geneoclinic, Dammam, Saudi Arabia

**Keywords:** Myelodysplastic syndrome, Cancer genomics

## Abstract

*SF3B1* gene mutations are prevalent in myelodysplastic syndrome (MDS) and define a distinct disease subtype. These mutations are associated with dysregulated genes and pathways, offering potential for novel therapeutic approaches. However, the aberrant mRNA alternative splicing landscape in *SF3B1*-deficient MDS cells remains underexplored. In this study, we investigated the influence of *SF3B1* gene alterations on the pre-mRNA splicing landscape in MDS cells using transcriptomic data from two independent MDS cohorts. we identified over 5000 significant differential alternative splicing events associated with *SF3B1* mutation. This work corroborates previous studies, showing significant enrichment of MYC activity and heme metabolism in *SF3B1* mutant cells. A key novel finding of this study is the identification of a gene expression signature driven by *SF3B1* mutations, centered on protein post-translational modifications. Notably, we discovered aberrant alternative splicing of the tumor suppressor gene *UBA7*, leading to significantly reduced gene expression. This dysregulation implicates UBA7 as a critical player in MDS pathogenesis. Importantly, the clinical relevance of this finding is underscored by the observation that low *UBA7* gene expression was associated with poor overall survival in chronic lymphocytic leukemia (CLL), another hematological malignancy with frequent *SF3B1* mutations. Furthermore, a similar association between low *UBA7* gene expression and poor survival outcomes was observed across multiple tumor types in the TCGA database, highlighting the broader implications of UBA7 dysregulation in cancer biology. These findings provide new insights into the mechanisms by which SF3B1 mutations reshape the pre-mRNA splicing landscape and drive disease pathogenesis in MDS. Furthermore, they underscore the potential of UBA7 as a biomarker to stratify SF3B1-mutant MDS and CLL patients, offering a refined approach for risk assessment and highlighting opportunities for targeted therapeutic interventions.

## Introduction

Myelodysplastic syndrome (MDS) is defined as a hematopoietic stem cells defect characterized by inefficient blood cell production and abnormal cell morphology. Clinically, it presents as peripheral blood low cell counts and carries a risk of progressing to acute myelogenous leukemia (AML). MDS affects 3 to 4 people per 100,000 in the United States, with its occurrence rising as individuals age^1^. Research over the past twenty years has shown that MDS is a diverse group of malignancies resulting from hematopoietic stem cell abnormal function, immune system dysregulation, altered apoptosis, and various aberrant genomic alternations^1–4^.

In recent years, significant progress has been made in characterizing the genetics landscape of MDS. Research has revealed that somatic mutations affecting genes with roles in the spliceosome occur in about 50% of MDS patients and that there is a distinct correlation between the type of spliceosome mutations and MDS disease stratification^5–7^. *SF3B1* gene alterations represent the most prevalent pre-mRNA splicing changes in MDS. The status of *SF3B1* gene is a key indicator for the presence of ring sideroblasts (RS), occurring in the majority of MDS patients who have ring sideroblasts (MDS-RS)^8,9^.

Genetic alterations in splicing factors are known to be early events in tumor development, and some researchers suggest that these mutations are not the primary drivers of the disease but rather add more fitness to tumor cells by facilitating pro-cancer changes^10–12^. Functional studies have shown that these gene changes are prevalent in signaling pathways and cellular processes that are dysregulated to trigger the acceleration of the disease, significantly influencing the clinical characteristics of MDS^13–15^. Moreover, research has identified specific genes and biological pathways that are prone to dysregulation in association with *SF3B1* mutations, presenting promising new therapeutic strategies^15^.

The pre-mRNA splicing landscape changes in *SF3B1* mutant MDS cells are not fully characterized due to the low number of reported cases of mutant and wild-type *SF3B1* MDS samples and/or the use of a population of cells that do not necessarily represent *SF3B1* deficient cells^16–18^.

Here, we performed a thorough and systematic analysis to assess the consequence of *SF3B1* alterations on the splicing landscape in MDS cells using larger cohorts. Key cellular processes and dysregulated pathways that supported prior findings were identified in CD34 + MDS cells. Genes that were prone to aberrant alternative splicing events were identified, and their gene expression and clinical relevance were assessed. Findings in this work were also evaluated in other independent cohorts with *SF3B1* mutation occurrences, such as chronic lymphocytic leukemia (CLL). The clinical relevance was also evaluated in other tumor contexts in the cancer genome atlas project (TCGA).

## Methods

### Patient cohorts

This research utilized open-access transcriptomic data from multiple independent MDS studies. The inclusion criteria were consistent initial cellular samples (CD34), samples from untreated MDS patients, and a paired-end NGS sequencing configuration to reduce potential technical noise. Two MDS studies met these criteria, and their transcriptomic data was found in public databases (GEO). Raw transcriptomic data for all two studies (n = 94) was retrieved from the GSE database. These studies are GSE114922 and GSE63569 collected in 2015, 2018, respectively^19,20^. Detailed RNA-seq sample information is provided in supplementary table S1 (Supplement S1). The data was accessed from the NCBI GEO database on January 19, 2024, using SRA tools. CLL (CLL-ES cohort N = 304) gene expression count matrix, and CLL patient’s clinical information was obtained from the International Cancer Genome Consortium (ICGC) open-access public repository at (https://dcc.icgc.org). The author did not have access to information that could recognize individual patients during or after data collection, as the data was already anonymized in the GEO and ICGC databases.

### Raw data processing and mapping

Raw sequencing data underwent quality control evaluation before being aligned to the hg38 reference genome using the STAR using 2-pass configuration to reliably and precisely identify novel splice junctions for differential splicing analysis^21^. The resulting BAM files were filtered, and duplicates were removed using Picard. Subsequently, read groups (RG) tags were added to sequencing reads using the tool Genome Analysis Toolkit (GATK)^22^. Sequencing reads within bam files containing (Ns) in their cigar string were split as they span splicing events in the data using the GATK tool.

### Percent-spliced-in (PSI) quantification and differential ASE analysis

Alternative splicing events (ASEs), such as inclusion/skipping of cassette exons (skipped exons, SE), alternative 5′/3′ splice site usage (A5SS/A3SS), intron retention (IR), are quantified using the percent-spliced-in (PSI) parameter, calculated by equation^23^. The formula for PSI calculation is as follows:

$$PSI = \frac{Inclusion\;Reads}{{Inclusion\;Reads + Exclusion\;Reads}}$$

ASE isoforms are identified based on the region size that is alternatively spliced, with the included isoform having a larger region than the excluded isoform. For mutually exclusive events such as the inclusion of mutually exclusive exons (MXE) and alternative first or last exons (AFE/ALE), the included isoform is defined by the shorter first intron. Isoform abundances are estimated using junction read counts, which do not require length normalization like exon body-aligned reads. For intron retention (IR) events, the included isoform’s abundance is determined using the mean depth of coverage across the intron, trimmed by 30%, as per IRFinder’s method^24^.

We identified two independent MDS cohorts with *SF3B1* mutations (N = 36) and those with wild-type *SF3B1* (N = 58). Differential splicing analysis was conducted by comparing SF3B1-mutant MDS samples to their wild-type counterparts. Differential ASE analysis was applied, utilizing a generalized linear model (GLM) based approach using the edgeR package^25^. In this analysis, estimated included and excluded isoform coverage matrices where columns representing samples and rows representing AS events were used as input for differential ASE analysis. The SF3B1 mutation status and the study cohort were described as factors in the GLM equation. AS events were defined as significant if they passed the significance cut off ( FDR < 0.05 and LogFC > −/ + 1.5).

### Differential gene expression

Gene expression count matrix was produced using the featureCounts tool^26^ with the GTF file from Gencode (GENCODE 46 (May 2024))^27^. The resulting count matrix was then used for differential gene expression analysis. DESeq2^28^ was used to find differentially expressed genes (DEGs) by contrasting *SF3B1*-mutant against *SF3B1*-wt MDS samples, considering a gene significant if it had an adjusted p-value below 0.05 and more than 50% fold change. This stringent cut-off produced lists of DEGs that were either upregulated or downregulated in MDS patients. Subsequently, Gene Set Enrichment Analysis (GSEA)^29^ was applied to the DEGs, identifying significant hallmarks. All plots were produced using R (Version 4.4.1).

### Survival analysis for TCGA and ICGA data

To evaluate the impact of *UBA7* gene expression in various tumors, we analyzed the TCGA cohort’s transcriptomic data and related clinical information. Normalized gene expression metrics for each cohort were obtained from the UCSC Xena project^30^. Cancer samples were categorized as High or Low based on the median expression value of UBA7 within each cohort. Survival curves were constructed using the survminer package and deemed significant if the log-rank test p-value was below 0.05. For CLL-ES gene expression data, the count data were normalized using the DESeq2 package and generated survival plots following the same methodology as with the TCGA data.

## Results

MDS cohorts raw data that met the inclusion criteria (methods) were identified and downloaded from the GEO database. The raw data were then supplied to the analysis pipeline. MDS samples (N = 94) with confirmed *SF3B1* genotype status were then identified and used for splicing analysis. The alternative splicing (AS) events were characterized, and splicing profiles were generated in *SF3B1*-mutant vs. *SF3B1*-wt in the transcriptome of MDS samples. Alternative splicing count estimation at the event level utilized both exon body and junction reads. A percentage spliced-in (PSI) was calculated for each splicing event, representing the proportion of reads supporting exon inclusion compared to the total number of reads^31^. Using a threshold of FDR < 0.05 and a log2 fold change above 1.5, a total of 5679 significantly differential AS events were identified (Supplement S2).

**Fig. 1:**
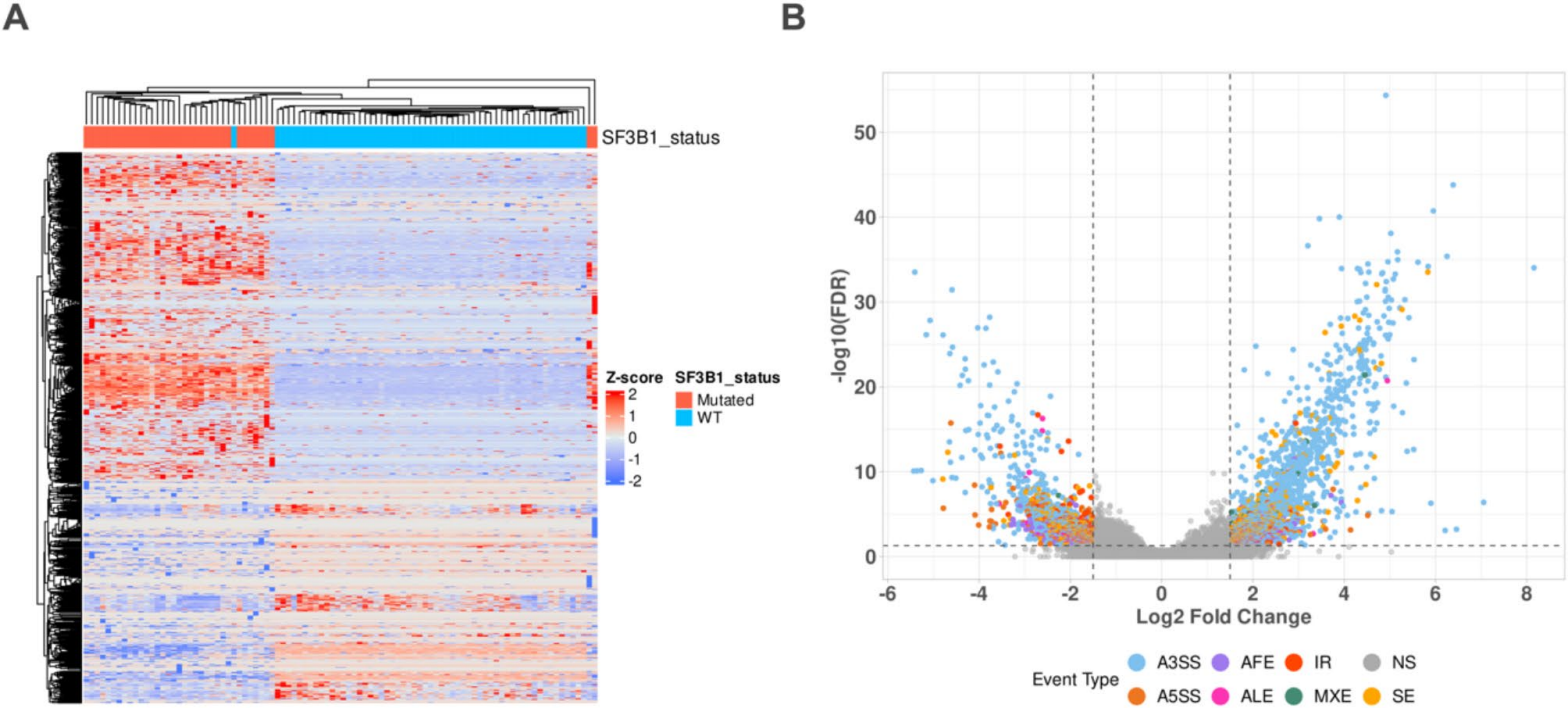
SF3B1 mutant MDS cells show a distinctive spliceosome landscape. (**A**) Unsupervised hierarchical clustering of significant AS events in SF3B1-mutant and SF3B1-WT MDS samples. AS events, PSI normalized values (Z-score) across samples are represented in rows. As events were considered significant if pass the predefined threshold (FDR < 0.05 and LogFC > −/ + 1.5). (**B**) Volcano plots showing the significance and effect size of identified AS events in SF3B1-mutant and SF3B1-WT MDS samples. Colors represent the type of AS event. (A3SS) alternative 3′ splice site usage, (SE) skipped exons, (A5SS) alternative 5′ splice site usage, (AFE) alternative first exons, (IR) intron retention, (ALE) alternative last exons, (NS) not significant.

**Fig. 2:**
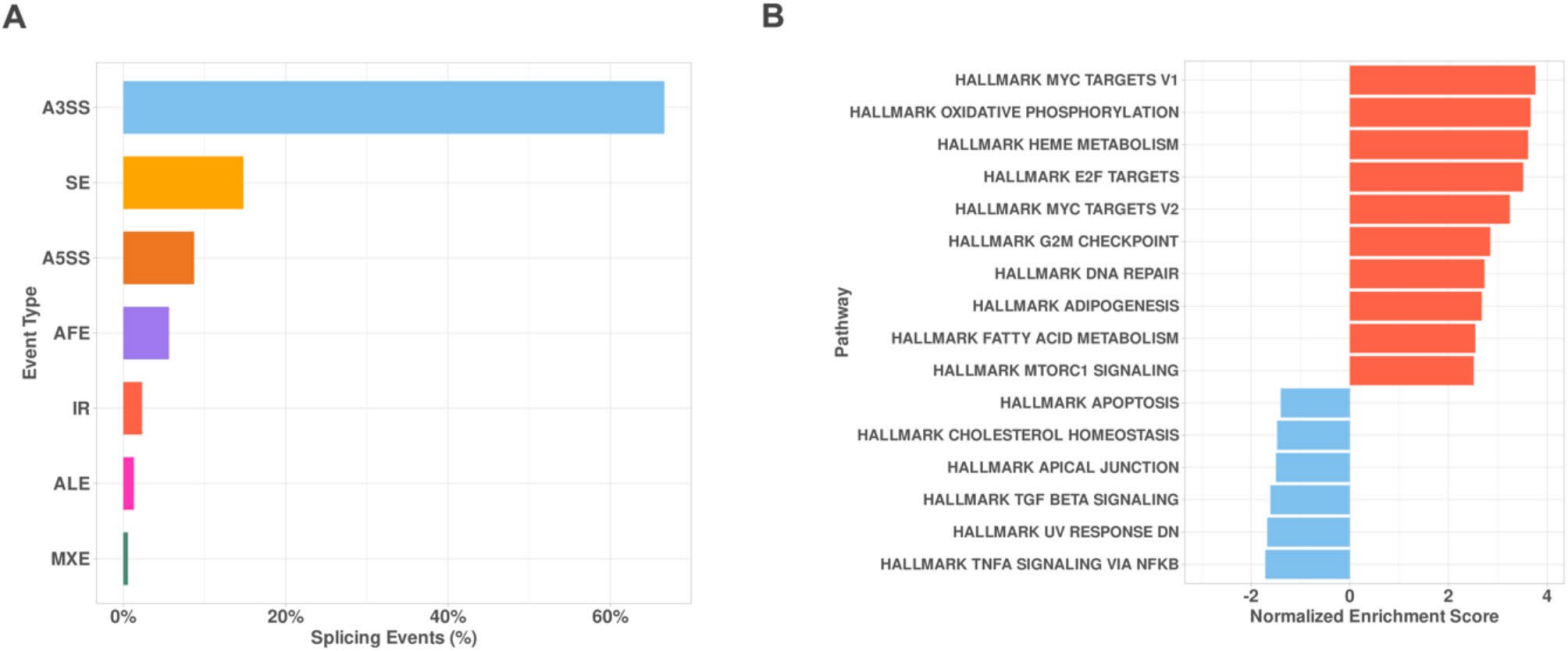
Splicing event distribution and pathway enrichment analysis in SF3B1-mutant MDS. (**A**) Bar plot depicting the distribution of alternative splicing (AS) event types identified in SF3B1-mutant MDS samples. The majority of events were annotated as alternative 3’ splice site (A3SS), followed by skipped exons (SE), alternative 5’ splice site (A5SS), alternative first exon (AFE), intron retention (IR), alternative last exon (ALE), and mutually exclusive exons (MXE). Percentages represent the proportion of each splicing event type relative to the total number of identified events. (**B**) Bar plot showing the pathway enrichment analysis based on Hallmark gene sets in *SF3B1*-mutant MDS samples. Pathways with positive normalized enrichment scores (NES) include MYC targets, oxidative phosphorylation, and heme metabolism, indicating significant upregulation. Pathways with negative NES, such as apoptosis, TGF-beta signaling, and TNF-alpha signaling, were downregulated. Results highlight the dysregulation of key pathways associated with *SF3B1* mutations.

These significant AS events were distinct and showed a differential pattern in the *SF3B1*-mutant vs. *SF3B1*-wt comparison (Fig. 1A). To have a holistic understanding of the distribution of these significant AS events, a volcano plot was generated, which shows the majority of AS events were increased in *SF3B1*-mutant MDS samples as expected since the splicing machinery is defective in these samples (Fig. 1B). The majority of these significant AS events were classified as alternative 3′ splice site usage (A3SS), which represents more than 60 percent, followed by skipped exons (SE), alternative 5′ splice site usage (A5SS), alternative first exons (AFE), intron retention (IR), alternative last exons (ALE), and inclusion of mutually exclusive exons (MXE) (Fig. 2A). Gene set enrichment analysis (GSEA) on differentially expressed genes identified an increased activity of MYC and heme metabolism pathways (Fig. 2B), which is consistent with previous reports^32–34^ (Supplement 3).

**Fig. 3:**
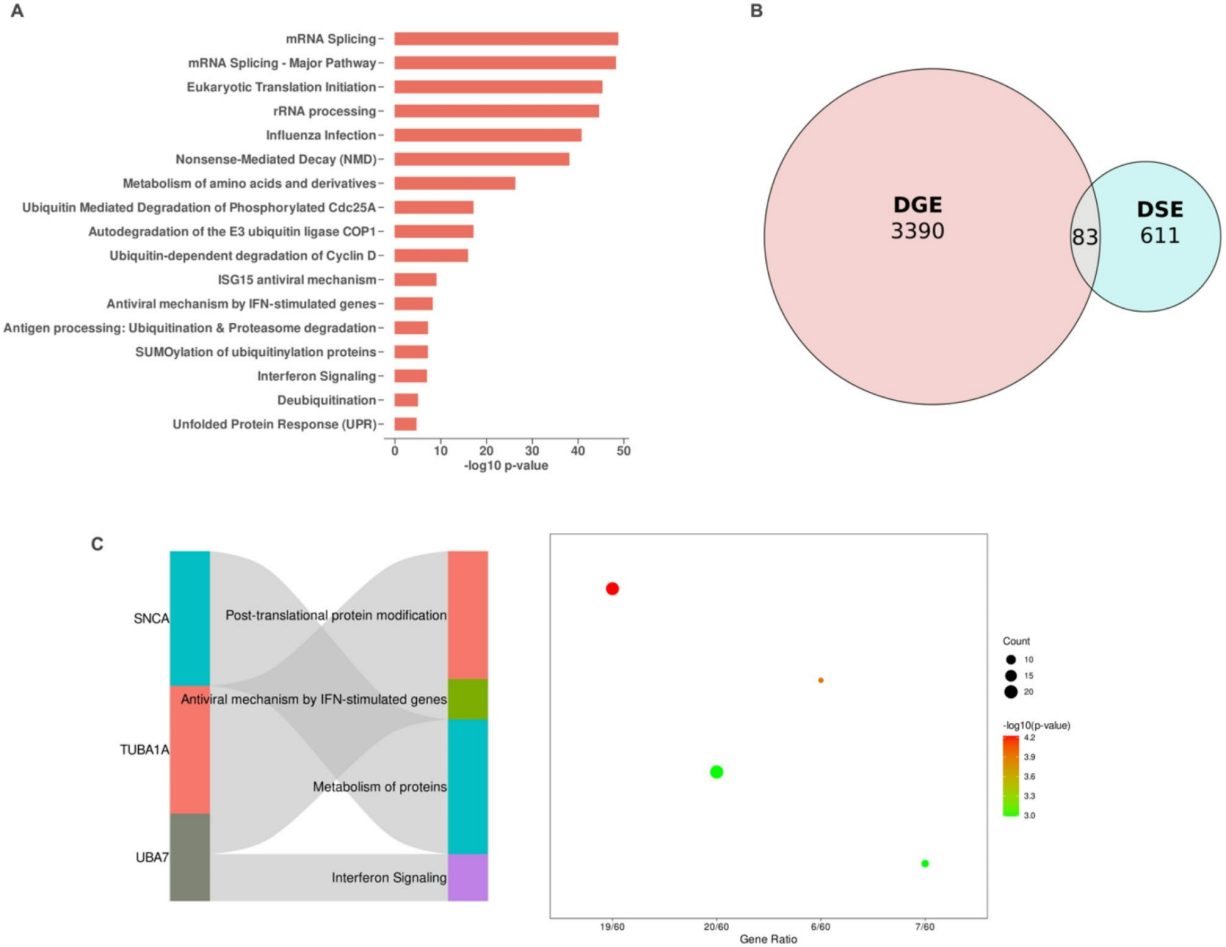
*SF3B1* mutations alter splicing and gene expression, highlighting pathways in protein modification and immune response. (**A**) Reactome pathway enrichment analysis of differentially spliced events (ΔPSI > 0.1, FDR < 0.05). The results highlight significant enrichment in pathways such as mRNA splicing, eukaryotic translation initiation, and post-translational protein modifications, underscoring the broad impact of *SF3B1* mutations on splicing and cellular processes. (**B**) Venn diagram illustrating the intersection of differentially expressed genes (DGE) (adjusted *p*-value < 0.05 and > 50% fold change) and differentially spliced events (DSE), identifying 83 genes that exhibit both significant changes in expression and splicing, further implicating their potential roles in *SF3B1*-driven pathogenesis. (**C**) Pathway analysis of the intersecting genes (DGE and DSE) reveals their involvement in key processes, including post-translational protein modifications, antiviral mechanisms by interferon-stimulated genes, and protein metabolism. Sankey plot highlights representative genes such as *UBA7, SNCA*, and *TUBA1A*, mapping their contributions to the associated pathways. The dot plot on the right shows the gene ratio, statistical significance (−log10 *p*-value), and enrichment magnitude for the top pathways.

To gain biological insights into the impact of genes exhibiting differential alternative splicing events (ASE) with an FDR < 0.05, we conducted a gene pathway enrichment analysis using the Reactome database^35^. As anticipated, genes associated with the mRNA splicing machinery and translation were predominantly affected by *SF3B1* mutations in MDS samples (Fig. 3A). Intriguingly, pathways related to protein post-translational modifications, including ubiquitin-mediated protein degradation, SUMOylation of ubiquitinylation proteins, and deubiquitination, were also significantly enriched. Additionally, we observed enrichment in pathways involved in interferon signaling and ISG15 antiviral signaling, highlighting the broader regulatory disruptions caused by defective splicing machinery.

To further refine the analysis and identify high-confidence genes, we intersected differential ASE genes (ΔPSI > 0.1, FDR < 0.05) with genes showing significant differential expression (adjusted p-value < 0.05 and > 50% fold change) (Supplement 4). This filtering process yielded 611 significant alternative splicing events (DSE) and 3,390 differentially expressed genes (DGE). The overlap between these two categories identified 83 genes that were both differentially spliced and differentially expressed (Fig. 3B). Reactome pathway enrichment analysis of this subset revealed predominant involvement in protein post-translational modifications, antiviral mechanisms mediated by interferon-stimulated genes, protein metabolism, and interferon signaling pathways (Fig. 3C).

**Fig. 4:**
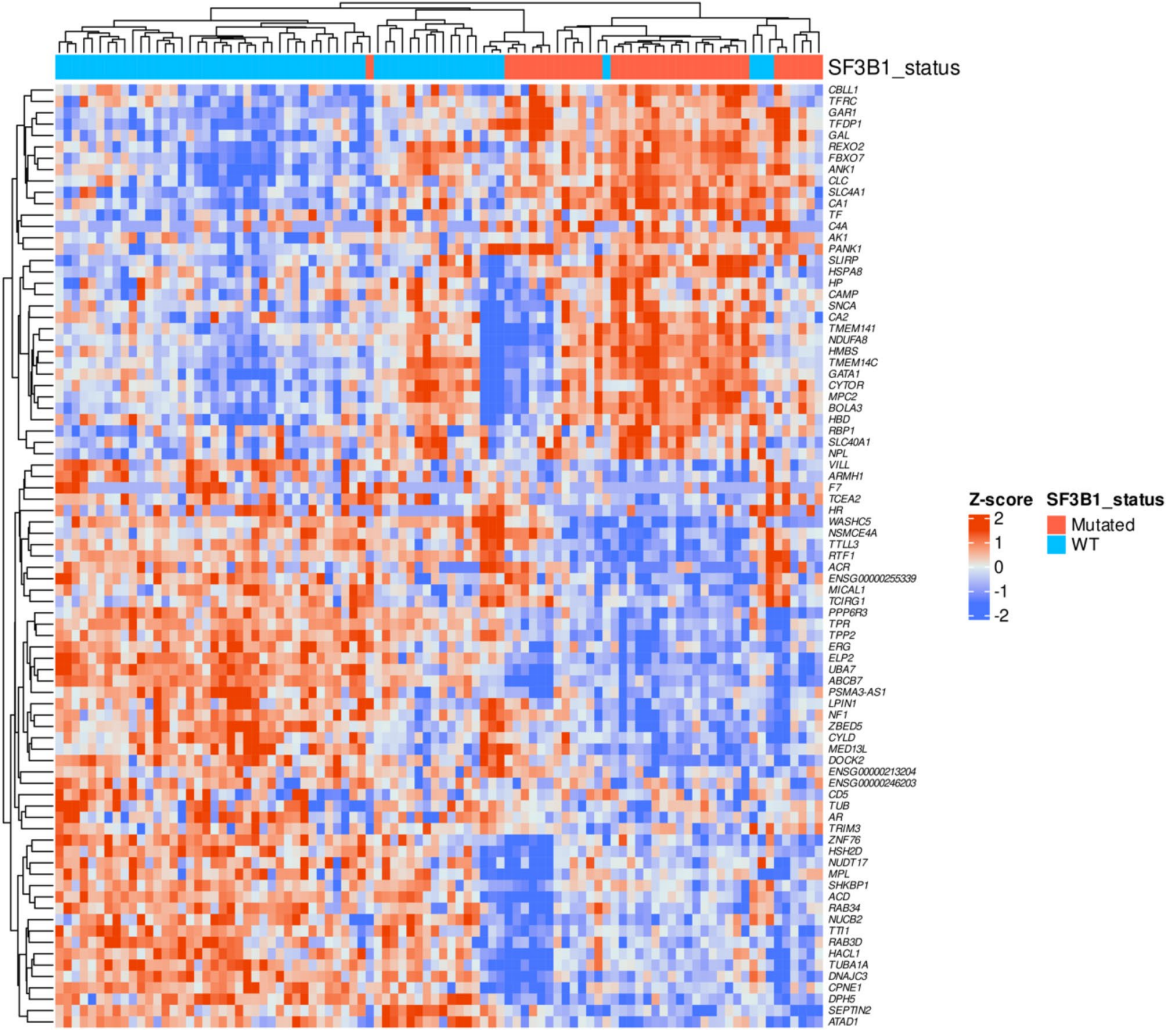
Transcriptomic landscape of *SF3B1*-mutant samples reveals distinct gene expression signatures. Hierarchical clustering heatmap showing the Z-score normalized expression of differentially expressed genes (DEGs) between *SF3B1*-mutant and wild-type (WT) samples. Each row represents a gene, and each column corresponds to a sample. Red indicates higher expression levels, while blue indicates lower expression levels. The *SF3B1* mutation status is denoted by the color bar above the heatmap (red for mutated and blue for WT). Genes are clustered based on their expression profiles, revealing distinct patterns of upregulated and downregulated genes associated with *SF3B1* mutations. These expression differences highlight the molecular impact of *SF3B1* mutations on the transcriptomic landscape of the samples analyzed.

Notably, these 83 genes exhibited a distinct expression profile that differentiated *SF3B1*-mutant MDS from *SF3B1*-wildtype MDS, suggesting a unique gene expression signature associated with *SF3B1* mutation status (Fig. 4). Specifically, the gene *UBA7* was involved in more than two pathways, underscoring its potentially profound impact and paving the way for a more focused investigation into its specific role in these processes.

**Fig. 5:**
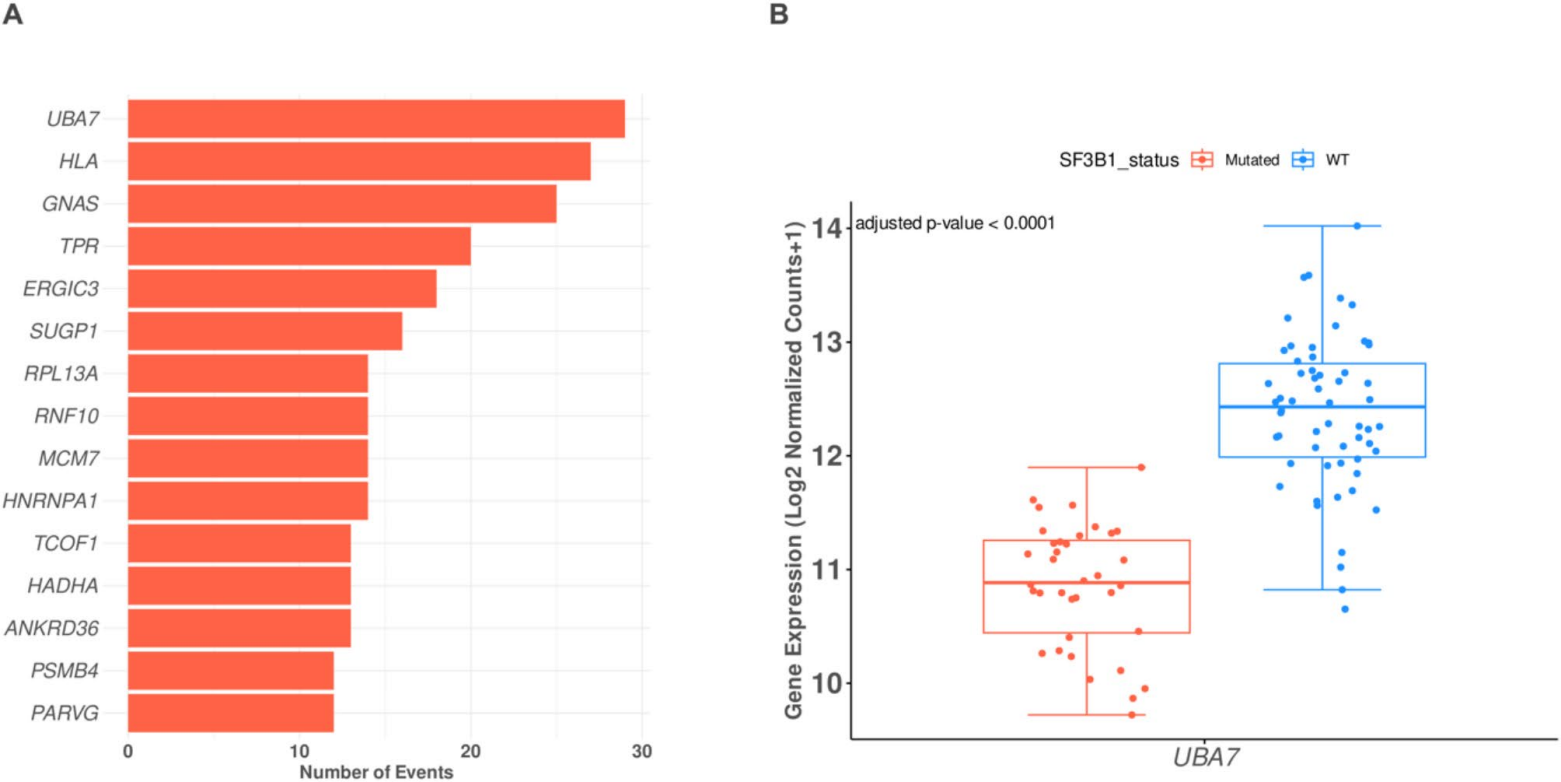
Spliceosome-mediated disruption of UBA7 in SF3B1-mutant samples. (**A**) Bar plot showing the top genes with the highest number of alternative splicing (AS) events in SF3B1-mutant samples. UBA7 demonstrates the greatest number of AS events, highlighting its susceptibility to splicing dysregulation in the context of SF3B1 mutations. (**B**) Boxplot illustrating the downregulation of UBA7 expression in SF3B1-mutant (red) compared to wild-type (WT) samples (blue). Gene expression is presented as log2-normalized counts, with a significant difference observed (adjusted *p*-value < 0.0001). This result suggests a functional impact of SF3B1 mutations on UBA7 expression, potentially contributing to disease pathogenesis.

Functional alterations associated with *SF3B1* mutations are likely due to abnormal splicing events in critical genes involved in tumorigenesis^36,37^. Approximately half of the genes linked with aberrant splicing events were subjected to nonsense-mediated decay, reducing gene and protein expression^32,38^. Therefore, this observation may affect key genes involved in cancer-protective mechanisms. To find genes subjected to an increased level of aberrant AS events, the number of AS events was counted for each gene and plotted as a horizontal bar plot (Fig. 5A). The results showed high AS events in genes such as *UBA7*, *HLA*, *RNF10, PSMB4, HNRNPA1, ERGIC3, SUGP1, and RPL13A*. To have a holistic overview of *UBA7* significant splicing events among other events a volcano plot was generated highlighting these events (Supplement 5A). Consistent with our previous findings (Fig. 3C), genes exhibiting a high frequency of alternative splicing events were predominantly associated with pathways related to protein post-translational modifications and interferon signaling. Among these genes, *UBA7* was significantly downregulated (Fig. 5B), which may have resulted from the observed alterations in splicing events that occurred during the maturation of mRNA produced from this gene**.**

To validate our findings, we analyzed the splicing landscape and expression profiles of key splicing machinery genes, including *SRSF2*, *U2AF1*, and *ZRSR2*, which are known to be frequently mutated in hematological malignancies. While we observed significant alternative splicing events in these genes, their frequency was notably low and did not substantially impact their gene expression profiles, suggesting that deregulated gene expression driven by aberrant alternative splicing is specific to *UBA7* (Supplement 5D–F, Supplements 8, 9, and 10). Furthermore, we identified significant alternative splicing events in previously established gene associated with *SF3B1*-mutant tumors, *BRD9*, which were accompanied by a marked reduction in gene expression (Supplement 5B and Supplement 6). These results are consistent with previous studies^32,36,39^, supporting a similar mechanism underlying the disruption of *UBA7* expression.

Ubiquitin-like modifier-activating enzyme 7 (UBA7) is a specialized E1-like enzyme that plays a pivotal role in the conjugation of interferon-stimulated gene 15 (ISG15). Most prior research on the UBA7/ISG15 signaling pathway in cancer has concentrated on lung and breast cancer and reported that *UBA7* gene expression was downregulated^40–43^. Combined with its association with various diseases, it underscores its biological importance. The high frequency of splicing events in *UBA7* observed in the MDS data highlights its potential impact on the regulation of protein post-translational modifications and interferon signaling. Additionally, the limited existing research on *UBA7* in the context of hematological malignancies and MDS in particular compared to other genes presents a unique opportunity to explore novel regulatory mechanisms and therapeutic targets. Therefore, the focus will be on UBA7 for the remainder of this paper to elucidate its functional implications and potential contributions to disease pathogenesis.

**Fig. 6:**
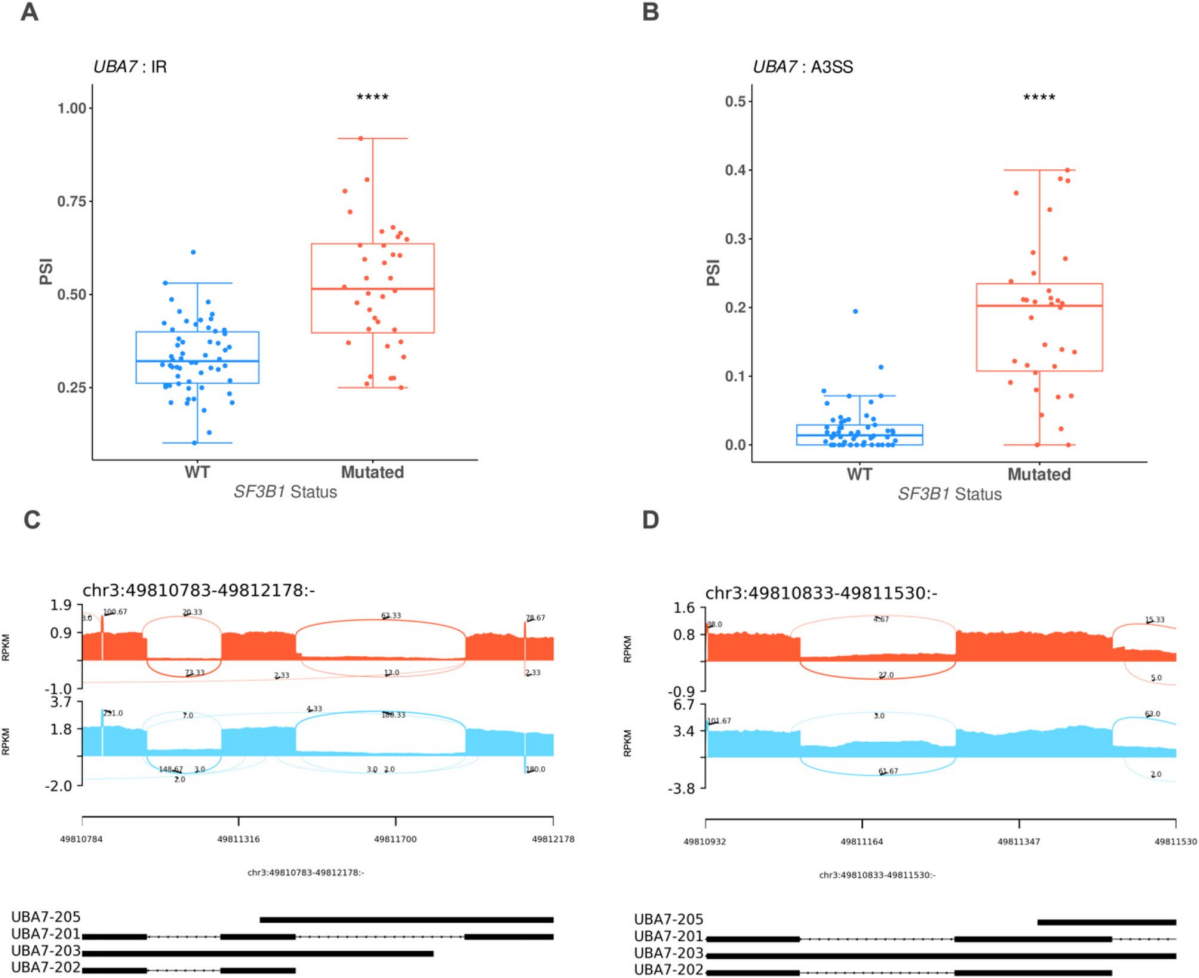
UBA7 alternative splicing events and gene expression are dysregulated in SF3B1 mutant MDS cells. (**A**) Boxplot showing the Percent-Spliced-In (PSI) values for intron retention (IR) events in UBA7, comparing wild-type (WT, blue) and *SF3B1*-mutant (red) samples. A significant increase in IR is observed in SF3B1-mutant samples (****p < 0.0001). (**B**) Boxplot displaying the PSI values for alternative 3’ splice site (A3SS) events in UBA7, with *SF3B1*-mutant samples (red) exhibiting significantly higher PSI values compared to WT (blue) (****p < 0.0001). (****FDR < 0.0001, ***FDR < 0.001, **FDR < 0.01, *FDR < 0.05). (**C**) Sashimi plot comparing RNA-seq read coverage and splicing patterns for *UBA7* between *SF3B1*-mutant (red) and WT (blue) samples, focusing on a specific genomic region. Numbers on the curved lines represent the average junction-spanning read counts across biological replicates, normalized using RPKM. The average number of junction-spanning reads is clearly higher in WT samples compared to *SF3B1*-mutant samples, indicating reduced splicing efficiency in the mutant group. (**D**) Sashimi plot comparing another genomic region of *UBA7* between *SF3B1*-mutant (red) and WT (blue) samples, showing similar trends with higher junction-spanning read counts in WT samples. The bottom black tracks represent the genomic localization of splicing events, providing a structural overview of exon–intron junctions. This figure highlights the spliceosome-mediated dysregulation of *UBA7* in *SF3B1*-mutant samples, characterized by increased intron retention, alternative 3’ splice site usage, and reduced splicing efficiency compared to wild-type samples.

The aberrant AS events changes associated with the *UBA7* gene were A3SS, MXE, and IR. In all these events, higher PSI values were associated with *SF3B1* mutations, selected AS events changes were plotted in Fig. 6A, B. In addition, the sashimi plots illustrate the RNA-seq read coverage and splicing patterns for *UBA7* in *SF3B1*-mutant and *SF3B1*-WT samples. Compared to WT samples, *SF3B1*-mutant samples exhibit increased intron retention and alternative 3’ splice site usage, highlighting significant splicing alterations in *UBA7* transcripts (Fig. 6C, D). These findings suggest that *SF3B1* mutations significantly impact the splicing and the expression of *UBA7*. Together, these findings suggest that *SF3B1* mutations contribute to MDS pathogenesis by disrupting the balance of protein post-translational modifications through aberrant alternative splicing events, consequently affecting key cancer barrier pathways.

Mutations in *SF3B1* are highly prevalent and significant in hematological malignancies^44,45^. In patients with chronic lymphocytic leukemia (CLL), *SF3B1* gene alterations occur in approximately 10–14% of cases. These mutations are linked to reduced overall survival rates in CLL patients^46^. To determine if the findings observed in MDS also apply to CLL, public gene expression data (n = 304) from the International Cancer Genome Consortium (ICGC) for CLL was analyzed.

To determine if *SF3B1* mutations in CLL are correlated with a similar reduction in *UBA7* gene expression as observed in MDS, we analyzed the ICGC CLL dataset. Although we observed a similar trend of *UBA7* downregulation in *SF3B1*-mutant CLL samples, the change was not statistically significant (Supplement 11A). Next, given that *SF3B1* mutations are known to co-occur with 11q deletions in CLL^47^, we investigated whether *UBA7* expression is associated with 11q deletions. Interestingly, we observed a slight upregulation of *UBA7* in CLL samples with 11q deletion, but this change was also not statistically significant (Supplement 11B). This observation suggests that *UBA7* gene expression changes may be specific to SF3B1 mutations, emphasizing the need for future functional studies to validate this finding and elucidate the underlying mechanisms. These observations may stem from the presence of non-functional *SF3B1* mutations, variations in allele frequencies, and the well-documented heterogeneity of the 11q deletion region in CLL, further underscoring the complex and multifaceted nature of CLL as a disease^48,49^.

**Fig. 7:**
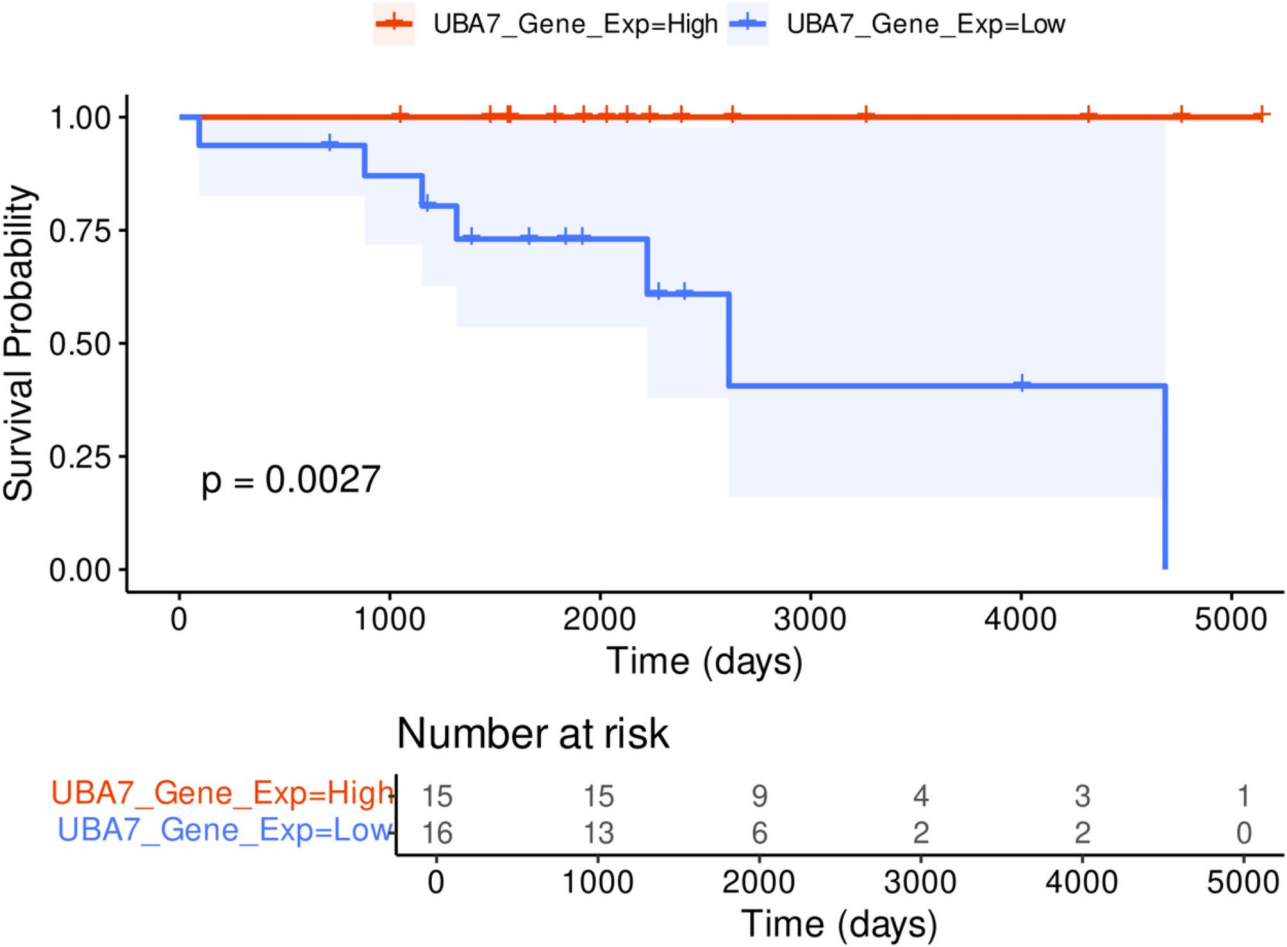
*UBA7* downregulation correlates with worse survival outcomes in chronic lymphocytic leukemia (CLL). Kaplan–Meier survival curves comparing overall survival probabilities between patients with high (red) and low (blue) *UBA7* gene expression levels. The analysis reveals that patients with low *UBA7* expression have significantly poorer survival outcomes compared to those with high expression levels (*p* = 0.0027). The shaded areas represent the 95% confidence intervals for each group. Below the survival curves, the "Number at risk" table indicates the number of patients remaining in each group at different time points.

Using the CLL ICGC associated clinical information, including survival data for CLL patients, allowed the investigation into the impact of *UBA7* gene expression on overall survival. CLL patients with *SF3B1* mutations were identified, and their gene expression data were imported. After normalizing this data, the median *UBA7* expression was used as a threshold to categorize patients into high and low UBA7 expression groups. Kaplan–Meier survival plots were then generated using the gene expression and clinical data. The analysis showed that CLL patients with low *UBA7* expression had significantly shorter survival times compared to those with high *UBA7* expression (Fig. 7), with the difference being statistically significant (log-rank p-value = 0.0027). *SF3B1* mutations significantly co-occur with 11q deletions, both of which are associated with aggressive disease and poor overall survival in CLL^47^. Our findings demonstrated that *UBA7* downregulation in *SF3B1*-mutant CLL is strongly linked to poor survival, suggesting its potential integration into prognostic models to enhance the prediction of disease progression. Furthermore, we were able to stratify CLL patients harboring *SF3B1* mutations based on the *UBA7* gene expression profile, providing a refined approach for risk assessment.

The Cancer Genome Atlas (TCGA) database represents an invaluable resource for studying cancer. To further examine the influence of *UBA7* gene expression on cancer patient survival, the integration of TCGA gene expression data with clinical information was utilized to construct Kaplan–Meier survival plots for each TCGA cohort (Supplement S12). Notably, six TCGA cancer cohorts exhibited significant results, showing that patients classified with low *UBA7* gene expression profiles have reduced overall survival rates, and this observation was statistically significant. These cohorts include Esophageal Carcinoma (ESCA) with a log-rank p-value of 0.024, Skin Cutaneous Melanoma (SKCM) with a log-rank p-value of less than 0.0001, Liver Hepatocellular Carcinoma (LIHC) with a log-rank p-value of 0.035, Breast Invasive Carcinoma (BRCA) with a log-rank p-value of 0.00029, Bladder Urothelial Carcinoma (BLCA) with a log-rank p-value of 0.0032, and Adrenocortical Carcinoma (ACC) with a log-rank *p*-value of 0.017.

These findings are consistent with previous observations in MDS and CLL, further supporting the role of UBA7 as a potential tumor suppressor. Our analysis identified a distinct gene expression signature associated with *SF3B1* mutations, centered on protein post-translational modification (PTM) and immune response pathways. Notably, *UBA7* exhibited a high frequency of alternative splicing events and low gene expression, implicating its dual role in PTM and interferon signaling. In conclusion, the consistent association between low *UBA7* expression and reduced survival across diverse cancer cohorts underscores the importance of *UBA7* as a key contributor to cancer progression and patient outcomes.

## Discussion

This work evaluated the impact of *SF3B1* gene alterations on the splicing landscape in the context of MDS. The results demonstrated that the splicing landscape in *SF3B1*-mutant MDS samples was significantly deregulated, impacting genes involved in protein post-translational modifications, as reflected in the identified gene expression signature. Among these affected genes, *UBA7* stood out due to its involvement in multiple biological pathways and its established role in carcinogenesis. These findings provide novel insights into how *SF3B1* mutant cells gain more fitness and participate in MDS pathogenesis and extend work done by Pellagatti et al.^19^.

The findings in this work with an increased sample size showed results that further support work done by Pellagatti et al. in terms of transcriptomic signatures associated with mutant *SF3B1* gene in pathways such as heme metabolism and mitochondrial metabolism (Fig. 2B). This work demonstrated that protein post-translational modification is disrupted in *SF3B1*-mutant MDS, as evidenced by the deregulation of the *UBA7* gene. This downregulation was driven by aberrant alternative splicing events, consistent with mechanisms observed in previous studies^36,39^ where *BRD9*, another gene subjected to spliceosome dysregulation, exhibited impaired gene expression.

*SF3B1* mutations play a multifaceted role in tumor pathogenesis. In numerous cancer cells, *SF3B1* mutations have been shown to disrupt various cellular processes, such as heme metabolism, immune surveillance, DNA damage maintenance, and Notch signaling^50–52^. Additionally, these mutations affect several cellular pathways, notably the Notch, NF-κB, and mitochondrial pathways^51^.

The splicing machinery has been reported to play diverse roles in cellular processes beyond its canonical function in splicing^53^. Notably, alternative splicing (AS) and protein post-translational modifications (PTMs) are known to work in concert, enabling precise, time- and cell-specific protein modifications essential for numerous cellular functions^54^. In support of this, studies have shown that the splicing machinery actively enhances the regulation of PTMs, and inefficient pre-mRNA splicing can disrupt multiple post-transcriptional regulatory processes, as demonstrated in *Caenorhabditis elegans*^55^. In our work, we observed significant gene expression deregulation in genes associated with protein post-translational modifications, supporting the notion that *SF3B1* mutations have roles extending beyond splicing. Specifically, UBA7, a critical member of the protein post-translational modification pathway, was notably impacted by this deregulation, providing novel insights into how *SF3B1*-mutant cells gain a survival advantage by exploiting these disrupted pathways. These findings underscore the intricate interplay between splicing and post-translational modifications in maintaining cellular homeostasis.

In this work, the *UBA7* gene was found to exhibit increased levels of alternative splicing (AS) events, which significantly impacted its gene expression and potentially its biological role. UBA7 plays a critical role in protein post-translational modification by catalyzing the attachment of ISG15 to cellular proteins through a process known as ISGylation. ISGylation is a reversible modification wherein the ubiquitin-like protein ISG15 is processed into a mature 15-kDa form, exposing its carboxyl-terminal LRLRGG motif. This motif facilitates the recognition and binding of ISG15 to lysine residues on target proteins, thereby influencing their function, stability, and localization^56^. The ISGylation process occurs via a cascade of three enzymatic steps: the E1 group of enzymes, such as UBA7 activates ISG15 by forming a high-energy thioester bond, the E2 group of enzymes transfers the activated ISG15, and E3 ligases mediate its covalent attachment to specific lysine residues on target proteins. Importantly, this modification is dynamically regulated by the deubiquitinase USP18, which removes ISG15 from proteins, ensuring precise control and cellular adaptability under varying conditions^57^

The role of ISGylation in cancer remains a topic of debate, with studies suggesting both tumor-suppressing and cancer-promoting effects. Elevated ISGylation has been shown to inhibit the camptothecin-dependent proteasome-mediated degradation of topoisomerase I in breast cancer cells, contributing to the efficacy of camptothecin as an anticancer chemotherapeutic agent^58^. On the other hand, several studies have demonstrated that ISGylation inhibits tumorigenesis by destabilizing proteins involved in growth regulation. For example, UBA7 targets key regulatory proteins such as cyclin D1 and PML-RARα for proteasomal degradation, suppressing the growth of human lung cancer cells^59,60^. Furthermore, free ISG15 has been shown to inhibit tumor growth when administered extracellularly, inducing the infiltration of NK cells into tumors in nude mice. Intracellular free ISG15 also enhances the 26S proteasome-dependent surface expression of MHC Class I complexes on breast cancer cells, further contributing to its tumor-suppressive role^61^. Recent findings by Wei Y. et al. reported that UBA1, a member of the E1 ubiquitin-activating enzyme family, is downregulated at both the mRNA and protein levels in myelodysplastic syndrome. Notably, their study revealed a tendency for SF3B1 mutations to co-occur with UBA1 downregulation, further highlighting the potential role of E1 enzyme family dysregulation in the pathogenesis of SF3B1-mutant MDS^62^.

Consistent with this notion, this study identifies a novel role for *UBA7* in MDS, and potentially CLL, as a tumor suppressor subjected to downregulation through disruptions in alternative splicing. Low *UBA7* gene expression was associated with poor survival as seen in CLL patients harboring *SF3B1* mutations, suggesting its potential use as a biomarker for adverse prognostic outcomes. These findings broaden our understanding of MDS pathogenesis, highlighting the critical role of the interferon-stimulated gene ISGylation network in tumor suppression.

The findings presented in this work are derived from comprehensive computational analyses, providing valuable insights into the dysregulation of *UBA7* in MDS and its potential roles in tumor biology. However, the absence of experimental validation represents a limitation that should be addressed in future research. Functional studies, including in vitro assays and in vivo models, are essential to confirm the involvement of UBA7 in ISGylation processes, immune evasion mechanisms, and tumor progression. These experiments could elucidate the molecular pathways regulated by UBA7 and its interactions with other key players in tumor biology. Moreover, evaluating UBA7’s functional relevance across diverse tumor types could reveal its broader implications as a pan-cancer target. Such studies would not only provide mechanistic validation but also enhance the translational significance of UBA7 as a potential therapeutic target and prognostic biomarker, paving the way for the development of targeted therapies and personalized treatment strategies in hematologic malignancies and beyond.

## Supplementary Information

Supplementary Information 1.

Supplementary Information 2.

## Data Availability

RNA-seq data used in this study are publicly available at GEO database and can be accessed on the following links: https://www.ncbi.nlm.nih.gov/geo/query/acc.cgi?acc=GSE63569, https://www.ncbi.nlm.nih.gov/geo/query/acc.cgi?acc=GSE114922.
