# Supplementary material for: Identification of UBA7 expression downregulation in myelodysplastic neoplasm with SF3B1 mutations
Source: Sci Rep. 2025 Mar 29;15:10856. doi: 10.1038/s41598-025-95738-9 (PMC11954878; doi:10.1038/s41598-025-95738-9)
Supplement: Supplementary file 2 — Supplementary Information 2. [file 41598_2025_95738_MOESM2_ESM.pdf]

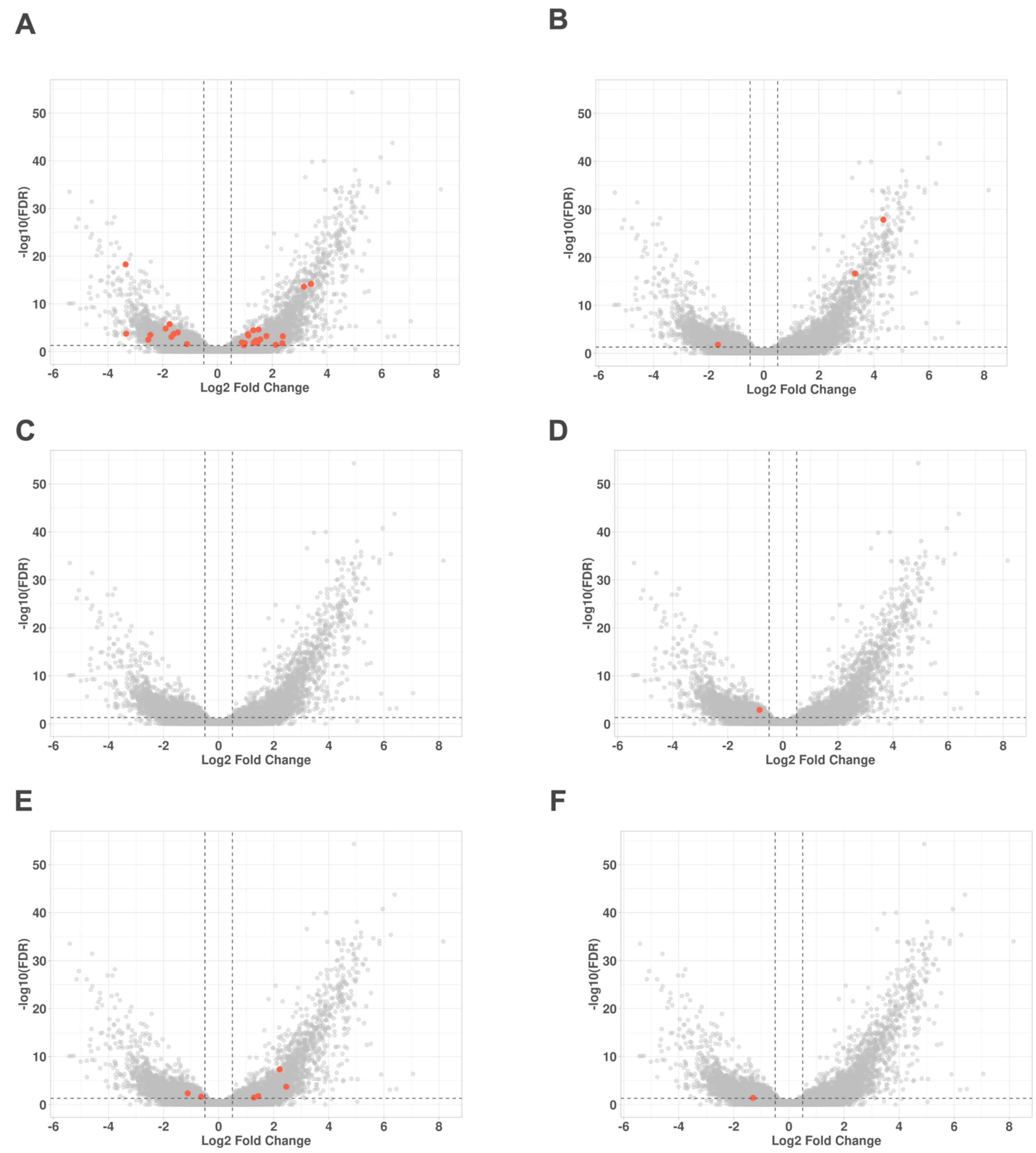

Supplement 5: Differential Splicing Events in Genes of Interest in MDS with *SF3B1* Mutation versus Wild-Type *SF3B1*.

Volcano plots depict splicing events identified when contrasting MDS with *SF3B1* mutation against MDS with wild-type *SF3B1*. Each point represents a splicing event, with red points indicating events with an FDR below 0.05. (A) *UBA7*, (B) *BRD9*, (C) *PPP2R5A*, (D) *SRSF2*, (E) *U2AF1*, and (F) *ZRSR2*. The x-axis shows the log2 fold change, and the y-axis indicates the -log10(FDR).

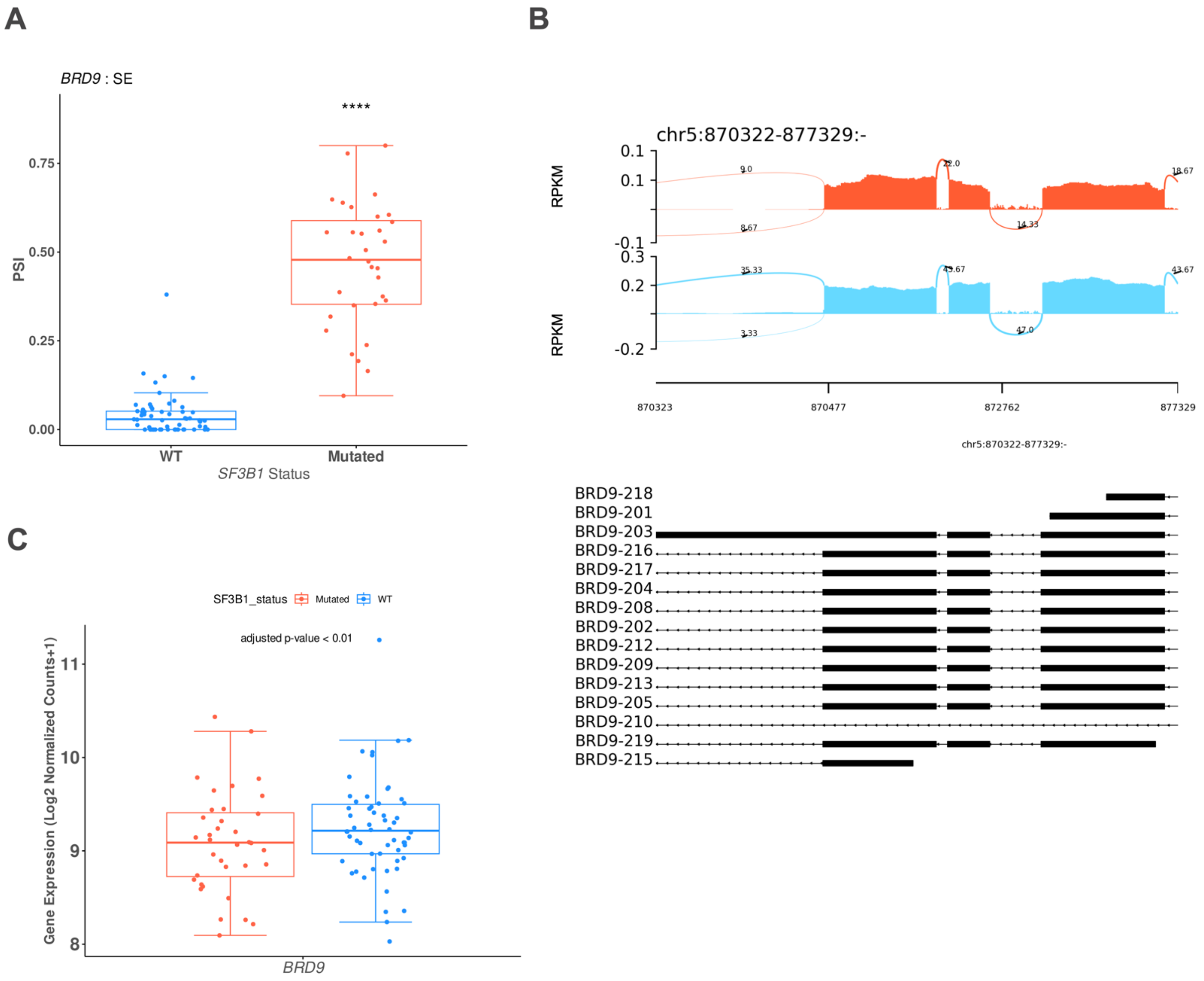

Supplement 6: Evaluation of splicing and expression changes in the *BRD9* gene across SF3B1-mutant and wild-type samples

(A) Boxplots showing the Percent-Spliced-In (PSI) values for selected splicing event skipped exon (SE) for *BRD9* between wild-type (WT, blue) and *SF3B1*-mutant (red) MDS samples. (****FDR < 0.0001, ***FDR < 0.001, **FDR < 0.01, *FDR < 0.05). (B) Sashimi plots comparing RNA-seq read coverage and splicing patterns of *BRD9* between WT (blue) and *SF3B1*-mutant (red) samples. Numbers on curved lines represent the average junction-spanning read counts for each group, normalized using RPKM. The genomic localization of splicing events is displayed below the plots, showing exon-intron structures and splice junctions for each gene. The sashimi plot does not appear to exhibit dramatic changes in junction-spanning read counts or splicing patterns between *SF3B1*-mutant and WT samples, unlike *UBA7*, where such differences were more evident. (C) Boxplots illustrating the log2- normalized expression levels of *BRD9* between WT (blue) and *SF3B1*-mutant (red) samples.

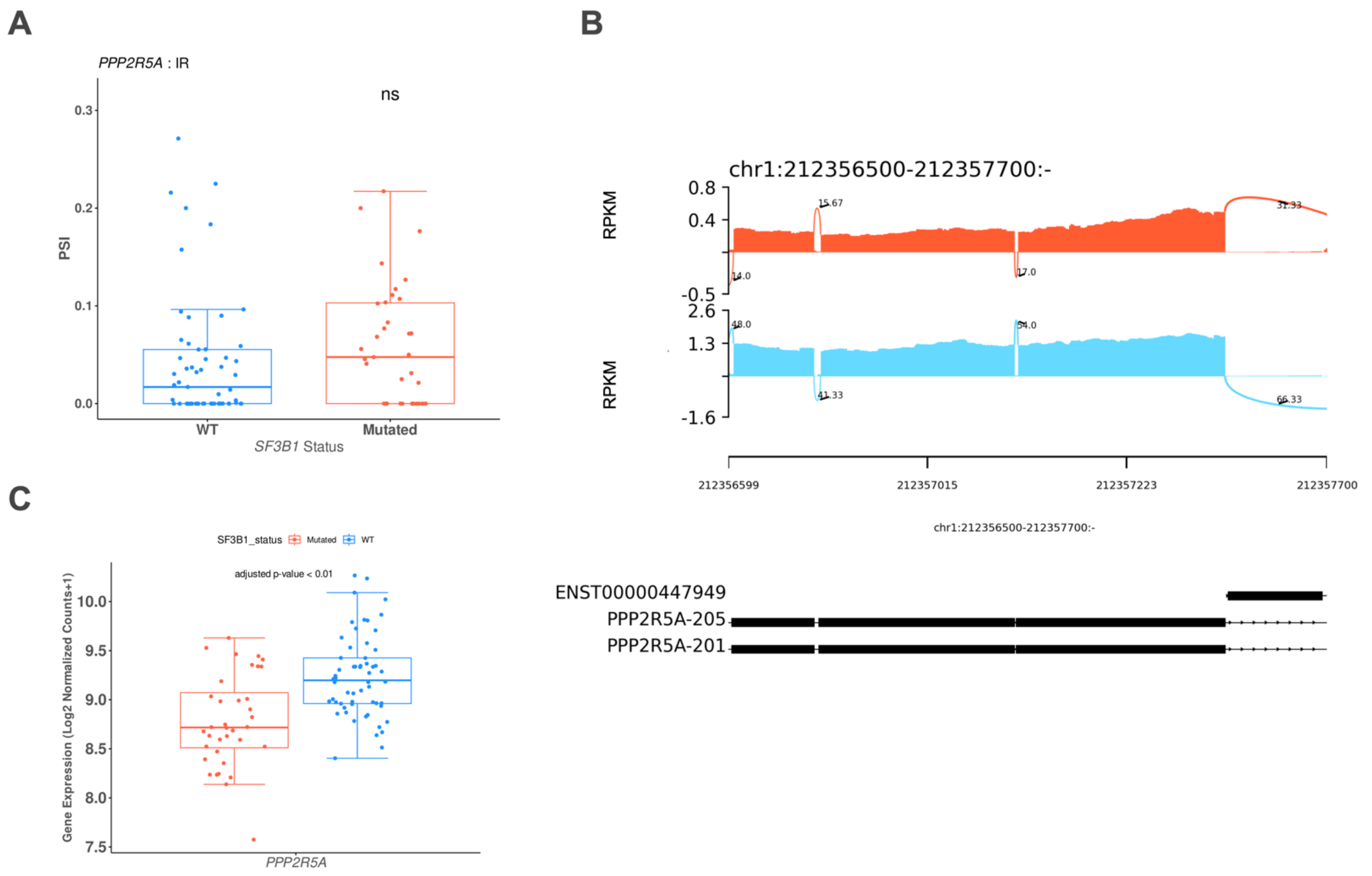

Supplement 7: Evaluation of splicing and expression changes in *PPP2R5A* gene across SF3B1-mutant and wild-type samples

(A) Boxplots showing the Percent-Spliced-In (PSI) values for selected splicing event intron retention (IR) for *PPP2R5A* between wild-type (WT, blue) and *SF3B1*-mutant (red) MDS samples. (****FDR < 0.0001, ***FDR < 0.001, **FDR < 0.01, *FDR < 0.05). (B) Sashimi plots comparing RNA-seq read coverage and splicing patterns of *PPP2R5A* between WT (blue) and *SF3B1*-mutant (red) samples. Numbers on curved lines represent the average junction-spanning read counts for each group, normalized using RPKM. The genomic localization of splicing events is displayed below the plots, showing exon-intron structures and splice junctions for each gene. The sashimi plot does not appear to exhibit dramatic changes in junction-spanning read counts or splicing patterns between *SF3B1*-mutant and WT samples, unlike *UBA7*, where such differences were more evident. (C) Boxplots illustrating the log2- normalized expression levels of *PPP2R5A* between WT (blue) and SF3B1-mutant (red) samples. *PPP2R5A* shows significantly low expression levels (adjusted p-value < 0.01).

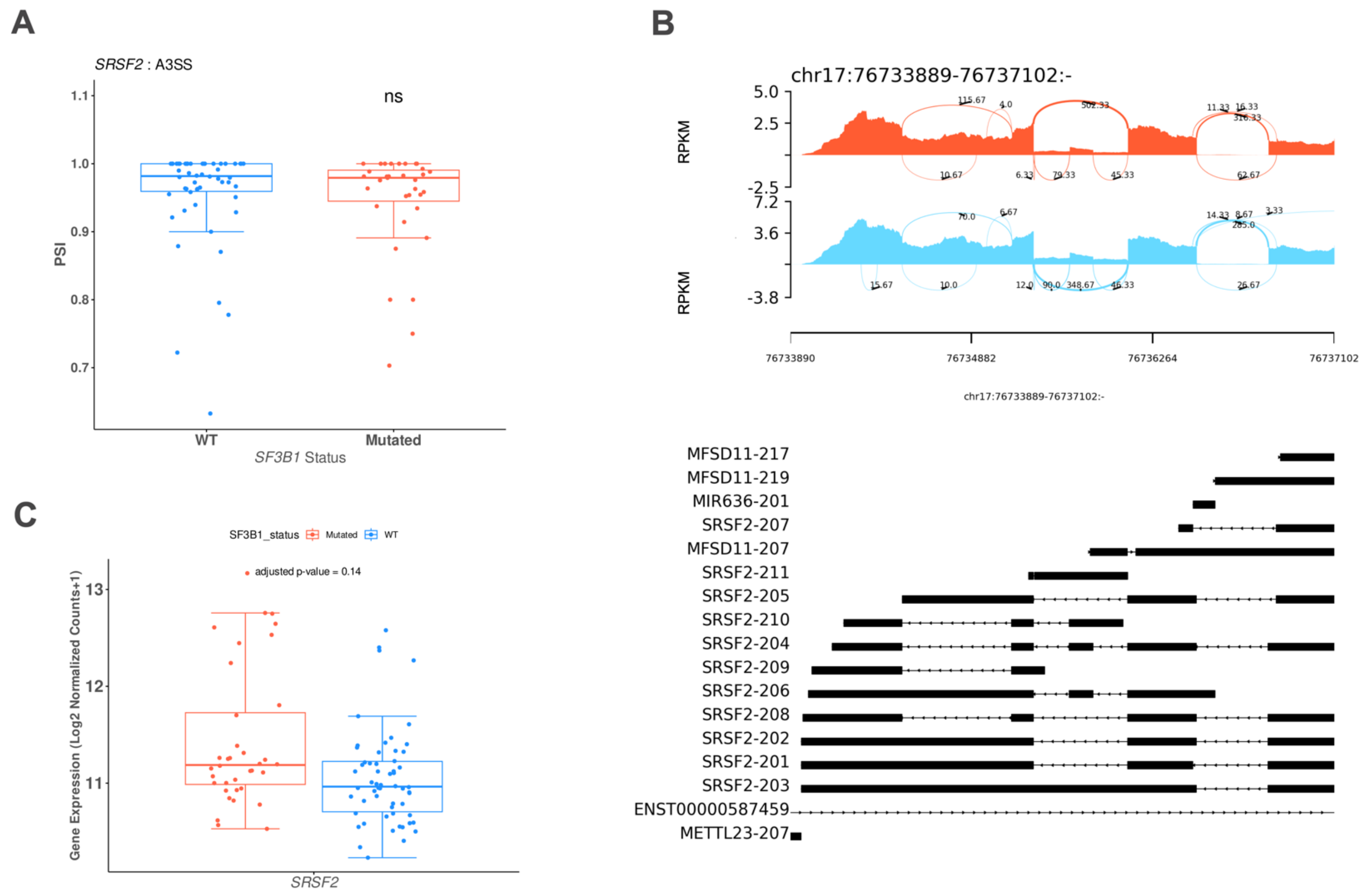

Supplement 8: Evaluation of splicing and expression changes in the *SRSF2* gene across SF3B1-mutant and wild-type samples

(A) Boxplots showing the Percent-Spliced-In (PSI) values for selected splicing event alternative 3' splice site (A3SS) for *SRSF2* between wild-type (WT, blue) and *SF3B1*-mutant (red) MDS samples. (****FDR < 0.0001, ***FDR < 0.001, **FDR < 0.01, *FDR < 0.05). (B) Sashimi plots comparing RNA-seq read coverage and splicing patterns of *SRSF2* between WT (blue) and *SF3B1*-mutant (red) samples. Numbers on curved lines represent the average junction-spanning read counts for each group, normalized using RPKM. The genomic localization of splicing events is displayed below the plots, showing exon-intron structures and splice junctions for each gene. The sashimi plot does not appear to exhibit dramatic changes in junction-spanning read counts or splicing patterns between SF3B1-mutant and WT samples, unlike UBA7, where such differences were more evident. (C) Boxplots illustrating the log2- normalized expression levels of *SRSF2* between WT (blue) and SF3B1-mutant (red) samples. *SRSF2* shows no significantly deregulated expression levels (adjusted p-value > 0.05).

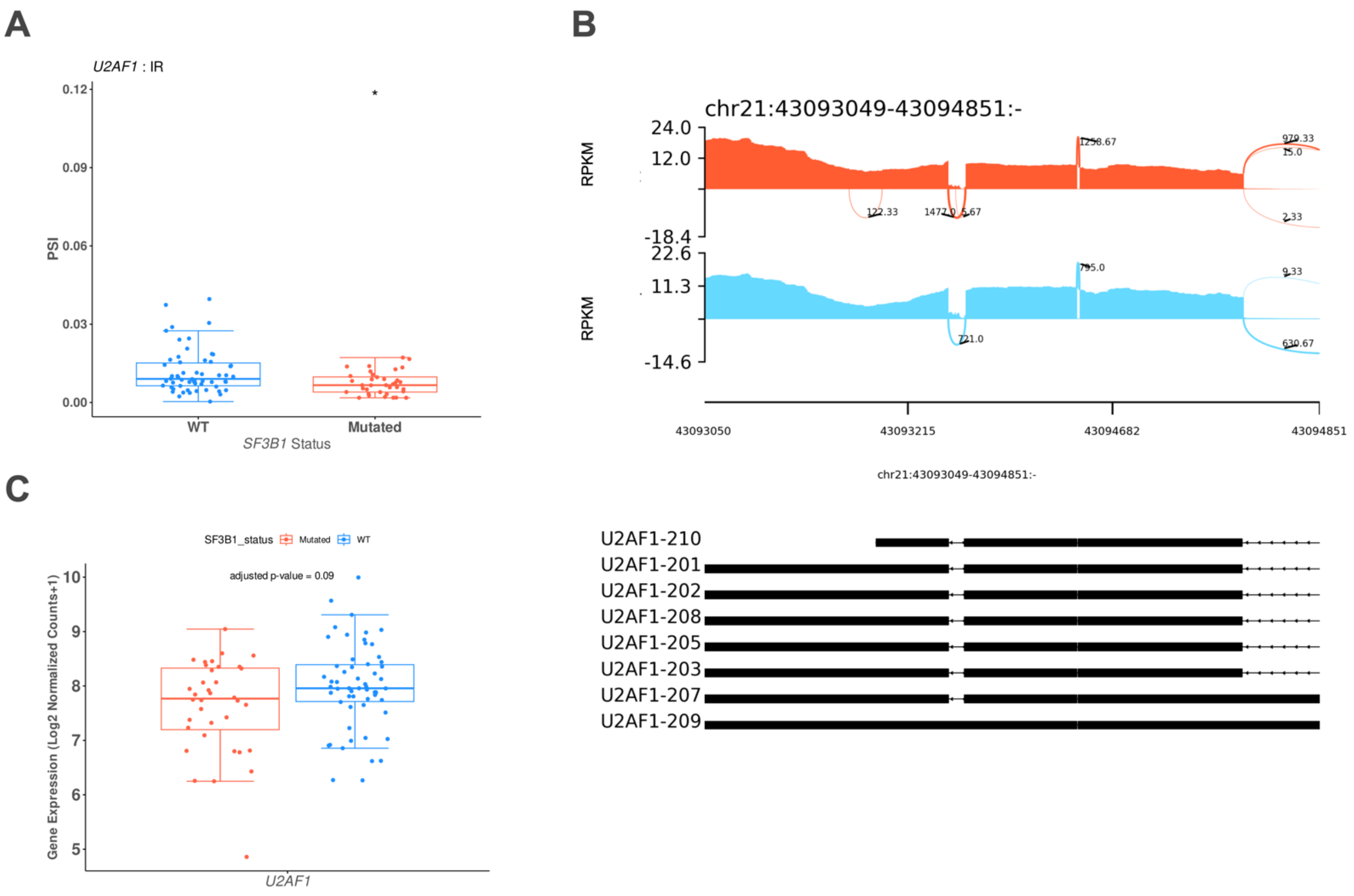

Supplement 9: Evaluation of splicing and expression changes in *U2AF1* gene across SF3B1-mutant and wild-type samples

(A) Boxplots showing the Percent-Spliced-In (PSI) values for selected splicing event intron retention (IR) event in *U2AF1* between wild-type (WT, blue) and *SF3B1*-mutant (red) MDS samples. (****FDR < 0.0001, ***FDR < 0.001, **FDR < 0.01, *FDR < 0.05). (B) Sashimi plots comparing RNA-seq read coverage and splicing patterns of *U2AF1* between WT (blue) and *SF3B1*-mutant (red) samples. Numbers on curved lines represent the average junction-spanning read counts for each group, normalized using RPKM. The genomic localization of splicing events is displayed below the plots, showing exon-intron structures and splice junctions for each gene. The sashimi plot does not appear to exhibit dramatic changes in junction-spanning read counts or splicing patterns between *SF3B1*-mutant and WT samples, unlike *UBA7*, where such differences were more evident. (C) Boxplots illustrating the log2- normalized expression levels of *U2AF1* between WT (blue) and *SF3B1*-mutant (red) samples. *U2AF1* shows no significantly deregulated expression levels (adjusted p-value > 0.05).

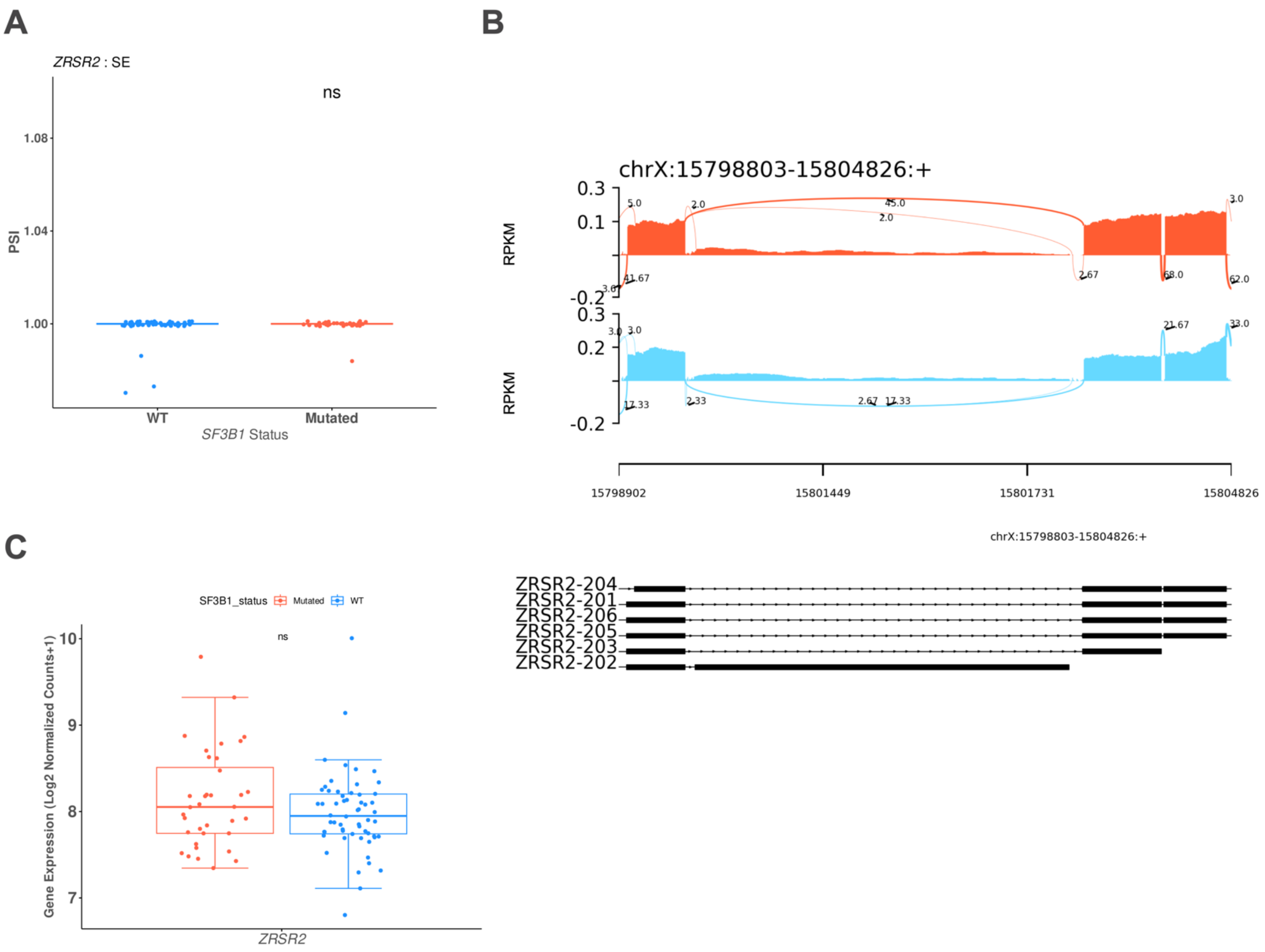

Supplement 10: Evaluation of splicing and expression changes in the *ZRSR2* gene across SF3B1-mutant and wild-type samples

(A) Boxplot showing the PSI values for skipped exon (SE) events in *ZRSR2* between WT (blue) and *SF3B1*-mutant (red) samples. No significant difference (ns) is observed. (B) Sashimi plot comparing RNA-seq read coverage and splicing patterns for ZRSR2 between WT (blue) and *SF3B1*-mutant (red) samples. The average junction-spanning read counts show no substantial differences between the groups. (F) Boxplot illustrating the log2- normalized expression levels of *ZRSR2* between WT (blue) and *SF3B1*-mutant (red) samples, showing no significant difference (ns).

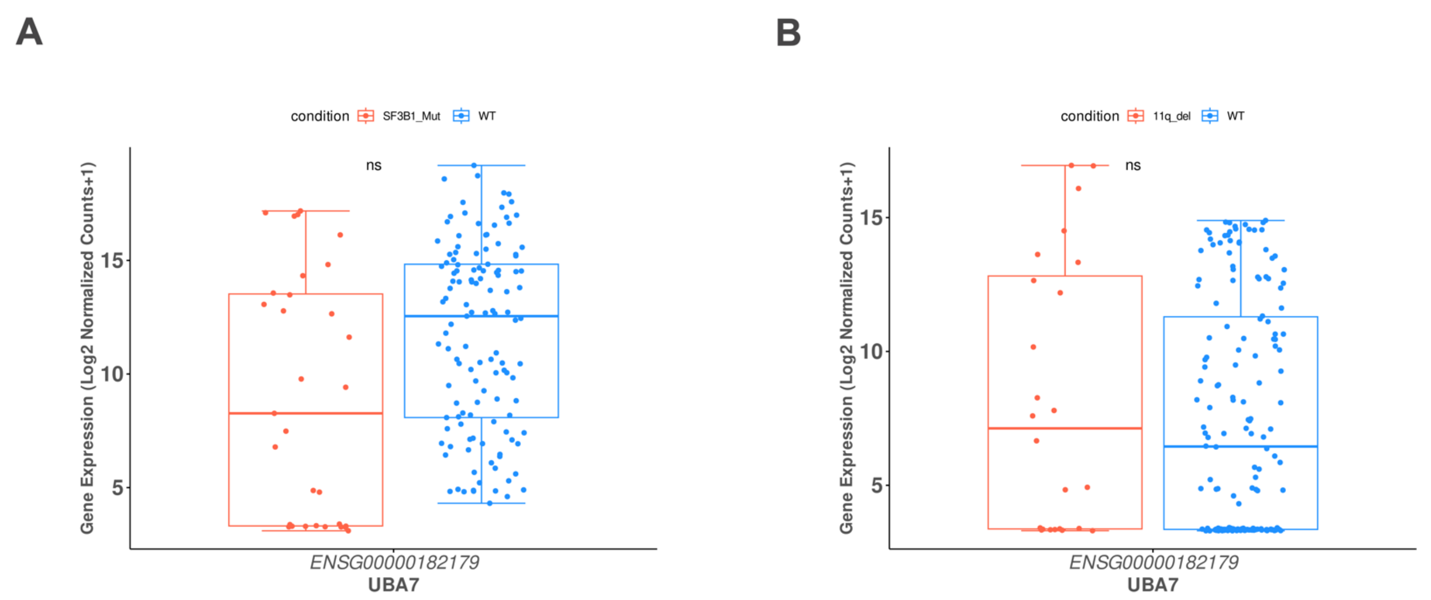

Supplement 11: *UBA7* gene expression levels across *SF3B1* mutation and 11q deletion conditions in CLL

(A) Boxplot comparing *UBA7* gene expression (log2-normalized counts) between *SF3B1*- mutant (red) and wild-type (WT) (blue) samples in CLL. Although a pattern similar to that observed in MDS, where *UBA7* is downregulated in *SF3B1*-mutant samples, is seen here, the difference in gene expression is not statistically significant (ns). (B) Boxplot comparing *UBA7* gene expression (log2-normalized counts) between samples with 11q deletion (red) and WT (blue). Similarly, no significant difference (ns) is observed, suggesting that *UBA7* expression is not substantially affected by the 11q deletion condition.

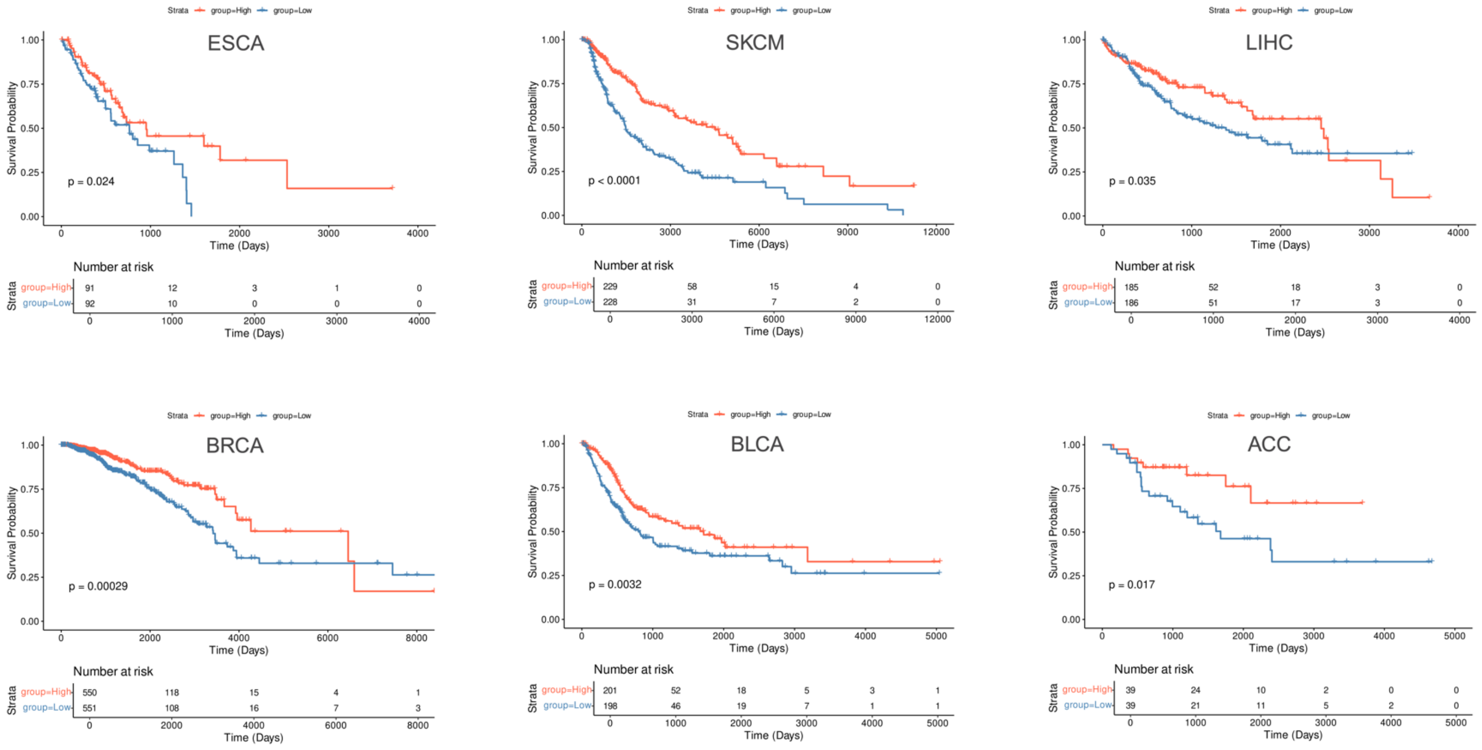

Supplement 12: *UBA7* low gene expression is associated with poor overall survival in multiple cancer cohorts on the TCGA database.

Kaplan-Meier survival curves demonstrating the association between *UBA7* gene expression levels (high vs. low) and overall survival across six cancer types: ESCA (Esophageal Carcinoma), SKCM (Skin Cutaneous Melanoma), LIHC (Liver Hepatocellular Carcinoma), BRCA (Breast Cancer), BLCA (Bladder Cancer), and ACC (Adrenocortical Carcinoma). This figure highlights the prognostic value of UBA7 gene expression levels across diverse cancer types and supports their potential use as biomarkers for patient stratification.
